# Differences and secular trends in childhood IQ trajectories in Guatemala City

**DOI:** 10.1016/j.intell.2020.101438

**Published:** 2020

**Authors:** Liina Mansukoski, Barry Bogin, J. Andres Galvez-Sobral, Luis Furlán, William Johnson

**Affiliations:** a Centre for Global Child Health, The Hospital for Sick Children, Toronto, Canada; b School of Sport, Exercise and Health Sciences, Loughborough University, Loughborough, UK; c UCSD/Salk Center for Academic Research and Training in Anthropogeny (CARTA), USA; d Centro de Investigaciones Educativas, Universidad del Valle de Guatemala, Guatemala City, Guatemala; e Centro de Estudios en Informática Aplicada, Universidad del Valle de Guatemala, Guatemala City, Guatemala

## Abstract

This study documents differences in childhood IQ trajectories of Guatemala City children, aged 6–15 years and born 1961–1993, according to school attended, height-for-age *Z*-scores (HAZ) and over time (Flynn effect). IQ data come from the Universidad del Valle de Guatemala Longitudinal Study of Child and Adolescent Development. IQ was measured using standardised tests from the Otis-Lennon Mental Ability Test-series. A multilevel model was developed to describe 60,986 IQ observations (level 1), in 22,724 children (level 2), in five schools representing students of different socioeconomic status (SES) (level 3). Average IQ trajectories differed by school. The difference in average IQ at age 11 years between the students of high and low SES schools was 28.7 points. A one-unit increase in HAZ was associated with a 1.42 (0.72, 2.11) unit higher IQ if HAZ was <0, this association was stronger in public compared to private schools. Conversely, one unit increase in HAZ was only associated with a 0.3 (0.001, 0.5) unit higher IQ if HAZ was ≥0. With each birth year increase, IQ at age 11 years increased by 0.14 (95% CI 0.12, 0.16) units, although this Flynn effect attenuated slightly across adolescence. We found no evidence of secular change in the inequality in IQ trajectories (according to school or HAZ). Shorter children from disadvantaged schools in Guatemala City have lower IQ than their taller and wealthier peers, possibly reflecting the damaging effects of poor early life environments both for linear growth and cognitive development.

## Highlights

• We modelled 60,986 IQ observations in 22,724 Guatemalan children aged 6–15 years, born 1961–1993, in five schools.
• Between-individual variance decreased with age; within-individual variance was higher in disadvantaged schools.
• There was a clear, graded difference in the modelled IQ trajectories across the schools.
• Being taller was associated with higher IQ, particularly in shorter children and disadvantaged schools.
• A secular trend in increasing IQ was observed but this Flynn effect did not differ across schools.

## 1 Introduction

Intelligence tests are commonly used to assess cognitive ability, and have utilitarian value because they are reasonably good predictors of grades at school, performance at work, and many other aspects of success in life (Nisbett et al., 2012). The Intelligence Quotient (IQ), estimated based on intelligence tests, has been positively associated with later life social and economic outcomes (Cheng & Furnham, 2012; Sorjonen, Hemmingsson, Lundin, Falkstedt, & Melin, 2012; von Stumm, Macintyre, Batty, Clark, & Deary, 2010), a lower risk of dementia, obesity, cardiovascular disease, some cancers, and mortality (Batty, Deary, & Gottfredson, 2007; Batty, Mortensen, Andersen, & Osler, 2005; Deary, Whiteman, Starr, Whalley, & Fox, 2004; Kanazawa, 2013; McGurn, Deary, & Starr, 2008; Whalley & Deary, 2001; Wraw, Deary, Gale, & Der, 2015). There is individual variation in the longitudinal stability of IQ scores over the growth period (Honzik, Macfarlane, & Allen, 1948; Hutchens, 1991; Mansukoski et al., 2019; Sternberg, Grigorenko, & Bundy, 2001; Tucker-Drob & Briley, 2014). After puberty, individual differences in intelligence plateau and remain relatively stable into old age, that is, individuals maintain their relative rank in IQ (Deary, 2014; Schalke et al., 2013),

Differences in IQ have been reported between socioeconomic status (SES) groups, schools, and by international population comparisons (Ceci & Williams, 1997; Engelhardt, Church, Paige Harden, & Tucker-Drob, 2019; Lynn & Vanhanen, 2012; Schwartz, 2015). Children of lower SES (e.g., according to parental education, income, and occupation) perform on average worse in cognitive tests than children from more privileged homes as early as 2 years of age in the UK, and this inequality continues throughout childhood and adolescence (von Stumm & Plomin, 2015). Differences in IQ related to the education a child receives and the school they attend have also been documented in high-income countries (Ceci & Williams, 1997; Ritchie & Tucker-Drob, 2018). Time spent in education has been estimated to increase IQ by 1 to 5 points per year (Ritchie & Tucker-Drob, 2018). Part of the education related differences in IQ are likely due to the background SES of the students themselves, and because lower SES students tend to attend poorer quality schools where differences may be exacerbated (Batty, Der, Macintyre, & Deary, 2006; Becker, Lüdtke, Trautwein, Köller, & Baumert, 2012; Brinch & Galloway, 2011; Ceci & Williams, 1997). Nonetheless, in many settings the school a child attends captures the community in which they live (e.g., housing type, income range, safety, ethnic make-up, social circle etc), and school-level differences in IQ are important to study as they reveal how entire communities of children are disadvantaged. In Latin America, a cross-sectional country comparison has reported large school related differences in both fluid intelligence and overall scholastic performance (Flores-Mendoza et al., 2015). To our knowledge, no previous study has investigated school-level differences in childhood to adolescent IQ trajectories in a low- or middle-income country (LMIC).

IQ is strongly related to characteristics of the individual. For example, there are reported associations between IQ and birth weight, brain anatomical structures and function, as well as personality traits such as creativity (Batey & Furnham, 2006; Deary, Penke, & Johnson, 2010; Kristensen et al., 2014). Height-for-age *Z*-scores (HAZ) are an excellent proxy of the cumulative exposures and environments people experience during their years of growth. It is partly for this reason that shorter height is associated with greater risk of negative later life health outcomes, including mortality, as well as lower human capital (NCD-RisC, 2016). There is a positive correlation between height and IQ of around *r* = 0.2 for adults (Teasdale, Owen, & Sorensen, 1991) and, similarly, children with short stature tend to score lower than their peers on cognitive ability tests (Bogin & MacVean, 1983; Wheeler, Bresnahan, Shephard, Lau, & Balk, 2004). A meta-analysis of studies in LMICs found that each unit increase in HAZ in infants aged under 2 years was associated with a + 0.24 SD increase in cognitive ability at ages 5–11, measured as any of the following: IQ, executive function, reasoning, language, or academic performance (Sudfeld et al., 2015). The association between HAZ in later childhood and adolescence, and IQ across childhood and adolescence is, however, not known.

As well as there being variation in IQ according to community and individual measures, there is variation over time (often proxied by birth year). It is well known that the average IQ test score has increased throughout the 20th century in various populations. This is known as the Flynn effect, named after James R. Flynn, or the secular rise in IQ scores (Trahan, Stuebing, Fletcher, & Hiscock, 2014; Williams, 2014). Studies show that there tend to be stronger gains in fluid than crystallised intelligence, as there have been greater gains in scores produced by nonverbal, performance-based measures like Raven's Progressive Matrices and Wechsler performance subtests (Trahan et al., 2014). More recently, there has been a reversal of the Flynn effect in many countries, the reason for which remains unknown (Dutton, van der Linden, & Lynn, 2016). Secular trends in IQ in LMICs have not been reported as widely as for high income samples (see Supplementary Materials in Pietschnig & Voracek, 2015). In Latin America, the Flynn effect has only been reported for Brazilian children aged 7–11 between 1930 and 2004, and Argentinian pupils and university students aged 13–24 between 1964 and 1998 (Colom, Flores-Mendoza, & Abad, 2007; Flynn & Rossi-Casé, 2012; Pietschnig & Voracek, 2015). Further, the extent to which differences in childhood-to-adolescent IQ trajectories have changed over time has not been investigated in Latin America.

The primary aim of this study was to investigate differences in IQ trajectories between ages 6–15 years by school in Guatemala. A secondary aim was to investigate the association of HAZ scores with IQ scores for each school. Given the study participants were born between 1961 and 1993, we also investigated the secular trend in IQ trajectories, and how any IQ differences between schools or by HAZ score might have changed over time. Guatemala is an important setting for this study because it 1) has one of the highest prevalence (48%) of under-5 stunting in the world and a high prevalence of stunting across family income levels which results in much height variation at all SES levels (MSPAS/INE/ICF, 2017; World Bank, 2018b), 2) is one of the most unequal societies in the world, with a Gini Index of 0.48 (World Bank, 2018a), and 3) over the last 70 years, the country has undergone major social, political, and economic upheavals, including a period of civil war between 1960 and 1996 (Lovell, 2010; Tomuschat, Lux De Coti, & Balsells Tojo, 1999), that may have influenced the secular trend in IQ by creating more or less inequality over time.

## 2 Subjects and methods

### 2.1 Study sample

The sample comprised 22,724 urban school children born between 1961 and 1993, aged 6 to 15 years, from Guatemala City, Guatemala. The children were participants in the Universidad del Valle de Guatemala (UVG) Longitudinal Study of Child and Adolescent Development (Bogin, Camacho de Paz, & MacVean, 2018). The study ran between years 1953 and 1999 in and around Guatemala City in seven schools, which (as in previous analyses) we grouped into five SES school groups (henceforth called schools) (Bogin et al., 2018):

• School 1 is a private institution charging high fees and is attended by some individuals of North American and European (primarily Spanish) heritage, but primarily by individuals of mixed heritage (self-identified ethnically as Ladinos).
• School 2 is comprised of two single-sex private Catholic schools (one for boys and one for girls) with moderate fees and attended by individuals of Spanish or Ladino ethnicity.
• School 3 comprises two co-educational Catholic private institutions with low fee or no fee and attended by individuals of Spanish or Ladino ethnicity.
• School 4 is a co-educational, state-run, non-sectarian institution with no fee, and attended by individuals of mostly Ladino ethnicity.
• School 5 comprises two co-educational, semi-urban, state-run, non-sectarian schools with no fee, and attended by individuals of indigenous Maya ethnicity.

Ladino or mixed heritage in the Guatemalan context refers to individuals of mixed Spanish and indigenous Maya heritage, but who identify with Spanish/European culture in terms of language (Spanish), social values, and religion (Bogin, Sullivan, Hauspie, & Macvean, 1989; Söchtig et al., 2015), Maya people identify with traditional indigenous language, behaviours, social values, and religion. The home language for many children in School 5 was Kaqchikel Maya. The school instruction language was Spanish for all pupils in the study. Schools were included in the study based on parental socioeconomic status as measured by the fees parents paid to the schools, their educational attainment, and occupation (Bogin et al., 2018). The socioeconomic composition of three of the study schools was investigated with a separate assessment in the 1980s (Bogin & MacVean, 1983). This analysis was conducted for a random sample of 672 families with children attending the study schools. The composite score had a range between 4 and 15 points. School 1 had a mean score of 12.2 (SD 3.4), for School 2 the average was 10.2 (SD 6.6) and for School 4 5.75 (SD 0.4) (Bogin & MacVean, 1983). Schools 3 and 5 were not assessed.

Children were automatically entered into the UVG study and approval for participation was collected from their parents. A team of trained investigators visited the study schools annually for data collection on all the students in attendance. The testing (IQ testing, anthropometry) was provided as a service to the study schools to give them information on child growth status. However, considering the socio-political context of the study period in Guatemala, it is possible that some parents, particularly those of Maya ethnicity, would not have felt confident to stop their children from participating in the study even if they so wished. The UVG Longitudinal Study was approved by the Guatemalan Ministry of Education, and the secondary analysis of these data has been approved by UVG and Loughborough University Human Participants Sub-Committee (Ref No: G13-P2).

### 2.2 IQ data

The current analysis only includes IQ data of individuals who took part in the study after 1976, as prior to this date, the study protocol for cognitive testing had not been standardised across schools, and different tests were used (Bogin et al., 2018). Post-1976, IQ was assessed solely using the Otis-Lennon Mental Ability Test (OLMAT) 1967 edition (Otis & Lennon, 1967). Briefly, the OLMAT is a multiple-choice pencil and paper test which measures verbal, quantitative, and spatial reasoning ability. The test was translated to Spanish at UVG, but not validated in Guatemala. The reference sample are US children. Test administration time was approximately 30 min, and the tests were administered in school years 1, 2, 4, 6, 7 and 9. In school year 1, children were 6–7 years old. Five different test levels were used, each test increases in difficulty: Elementary I Level Form J (school year 1), Elementary I Level Form K (school year 2), Elementary II Level Form K (school year 4), Intermediate Level Form J (school years 6 and 7), and Advanced Level Form J (school year 9). Each test consists of 80 items, 46 of which are verbal, 17 symbolic, and 17 figural. Item content includes word meanings, verbal similarities, quantitative reasoning, and reasoning by analogy (El-Korashy, 1995). The overall scores were converted to age-standardised IQ values using conversion tables provided by the test manufacturers. In total, there were 60,986 IQ observations, with an average of 2.7 observations per child (range 1–6).

### 2.3 Other data

Sex was coded as a categorical variable with 1 = male (referent), and 2 = female. Date of birth and date of measurement were coded as decimal dates. Age was calculated as decimal date of birth subtracted from decimal date of measurement. Height (cm) was measured to closest millimetre in standing position using a stadiometer. Height for age *Z*-scores were calculated using the WHO reference data(WHO, 2007). Stunting was defined as a categorical variable with HAZ ≥ −2 coded as non-stunted (0) and HAZ < −2 coded as stunted (1).

### 2.4 Statistical analyses

Descriptive statistics were produced stratified by school.

To explore clustering within the data, we created a variance components model with measurement occasion at level one, individuals at level two, and school at level three. Following this analysis, we developed a multilevel general linear regression model (see Eq. 1). The benefit of this approach, compared to a traditional linear regression with school incorporated as an independent variable, is that it correctly accounts for the clustering present in the data and leads to more accurate estimates (Goldstein, 2011; Goldstein, Browne, & Rasbash, 2002).

The dependent variable was IQ and the independent variables were age (centered at age 11 years), birth year (centered at 1980), HAZ (centered at 0), and sex (females vs male). In a first step of model building we considered and explored potential non-linearity using restricted cubic splines. Observed non-linearity in the IQ-age association was approximated using a quadratic polynomial. Non-linearity in the IQ-HAZ association was approximated using a linear spline with one knot, producing one term for values <0 and one term for values ≥0. There was no evidence of non-linearity in the IQ-birth year association, so birth year was included as a linear term. In the second step of model building we considered all potential two-way interactions but only found strong evidence (*p* < .001) to retain a birth year X age interaction term. In the third step of model building, the constant, age, and age^2^ terms were allowed to have random effects at level two & three, thereby allowing each individual and school to have their own trajectory. The HAZ < 0 term was allowed to have a random effect at level three, thereby allowing its effect to differ between the schools. There was no strong evidence that any of the other variables (e.g., birth year) required a random effect at level three. Finally, the level one (i.e., within-person) variance was allowed to differ across the schools.

We present our results with *P*-values to indicate combability with null distributions but did not perform traditional null-hypothesis testing. The estimates were evaluated by considering each estimate and its preciseness (95% confidence intervals). This follows the guidance from the American Statistical Association and the practice of leading epidemiology journals (Wasserstein & Lazar, 2016).

(1) $${\mathrm{IQ}}_{\mathrm{ijk}}=\beta_{0\mathrm{jk}}+\beta_{1\mathrm{jk}}{\mathrm{Age}}_{\mathrm{ijk}}+\beta_{2\mathrm{jk}}{\mathrm{Age}}_{\mathrm{ijk}}^{2}+\beta_{3}{\mathrm{Birth year}}_{\mathrm{jk}}+\beta_{4}{\mathrm{Age}}_{\mathrm{ijk}}\;X\;{\mathrm{Birth year}}_{\mathrm{jk}}+\beta_{5k}\mathrm{Height Zscore if}<0_{\mathrm{ijk}}+\beta_{6}\mathrm{Height Zscore if}\geq 0_{\mathrm{ijk}}+\beta_{7}{\mathrm{Sex}}_{\mathrm{jk}}+\varepsilon_{0\mathrm{ijk}}\mathrm{school}\;1_{k}+\varepsilon_{1\mathrm{ijk}}\mathrm{school}\;2_{k}+\varepsilon_{2\mathrm{ijk}}\mathrm{school}\;3_{k}+\varepsilon_{3\mathrm{ijk}}\mathrm{school}\;4_{k}+\varepsilon_{4\mathrm{ijk}}\mathrm{school}\;5_{k}$$

$$\beta_{0\mathrm{jk}}=\beta_{0}+v_{0k}+\mu_{0\mathrm{jk}}$$

$$\beta_{1\mathrm{jk}}=\beta_{1}+v_{1k}+\mu_{1\mathrm{jk}}$$

$$\beta_{2\mathrm{jk}}=\beta_{2}+v_{2k}+\mu_{2\mathrm{jk}}$$

$$\beta_{5k}=\beta_{5}+v_{5k}$$

$$\left[\begin{array}{c} v_{0k}\\ v_{1k}\\ v_{2k}\\ v_{5k} \end{array}\right]\sim N\left(0,\Omega_{v}\right):\Omega_{v}=\left[\begin{array}{c} \sigma_{v0}^{2}\\ 0\\ 0\\ 0 \end{array}\begin{array}{c} \sigma_{v1}^{2}\\ 0\\ 0 \end{array}\begin{array}{c} \sigma_{v2}^{2}\\ 0 \end{array}\begin{array}{c} \sigma_{v5}^{2} \end{array}\right]$$

$$\left[\begin{array}{c} \begin{array}{c} \mu_{0\mathrm{jk}}\\ \mu_{1\mathrm{jk}} \end{array}\\ \mu_{2\mathrm{jk}} \end{array}\right]\sim N\left(0,\Omega_{\mu }\right):\Omega_{\mu }=\left[\begin{array}{ccc} \sigma_{\mu 0}^{2} & & \\ \sigma_{\mu 01} & \sigma_{\mu 1}^{2} & \\ \sigma_{\mu 02} & \sigma_{\mu 12} & \sigma_{\mu 2}^{2} \end{array}\right]$$

$$\left[\begin{array}{c} \begin{array}{c} \begin{array}{c} \varepsilon_{0\mathrm{ijk}}\\ \varepsilon_{1\mathrm{ijk}} \end{array}\\ \varepsilon_{2\mathrm{ijk}} \end{array}\\ \varepsilon_{3\mathrm{ijk}}\\ \varepsilon_{4\mathrm{ijk}} \end{array}\right]\sim N\left(0,\Omega_{\varepsilon }\right):\Omega_{\varepsilon }=\left[\begin{array}{c} \begin{array}{c} \sigma_{\varepsilon 0}^{2}\\ 0\\ 0 \end{array}\\ 0\\ 0 \end{array}\begin{array}{c} \begin{array}{c} \sigma_{\varepsilon 1}^{2}\\ 0 \end{array}\\ 0\\ 0 \end{array}\begin{array}{c} \begin{array}{c} \sigma_{\varepsilon 2}^{2} \end{array}\\ 0\\ 0 \end{array}\begin{array}{c} \sigma_{\varepsilon 3}^{2}\\ 0 \end{array}\begin{array}{c} \sigma_{\varepsilon 4}^{2} \end{array}\right]$$

Where,

• *IQ*_*ijk*_ is the outcome at visit *i* of individual *j* in school *k*
• *β*_0−7_ are fixed effects
• *v*_0−2*k*_ and *v*_5*k*_ are random effects at level three (i.e., school)
• *μ*_0−2*jk*_ are random effects at level two (i.e., individual)
• *e*_0−4*ijk*_ are random effects at level one (i.e., visit) for each school
• All random effects are assumed to follow a normal distribution, with a mean of zero, and a variance (e.g., *σ*_*v*0_^2^).
• *σ*_*μ*01_, *σ*_*μ*02_, *σ*_*μ*12_ capture the covariances between the random effects at level two. Covariances at level one and three were constrained to be zero.

Using the final model, one figure was created showing how the level 2 (i.e., between-child) variance changes across the age range, one figure was created showing the IQ trajectories according to school, one figure was created illustrating differences in IQ trajectories according to birth year, and one figure was created showing the density distribution of HAZ and its relationship with IQ (for each school).

All procedures were performed in Stata 15 (StataCorp LP, College Station, TX, USA). The command runmlwin was used for the multilevel models (Leckie & Charlton, 2012).

## 3 Results

There were large differences in both IQ and prevalence of stunting between schools (Table 1). Across all observations, children attending School 1 had an average IQ of 107.2 compared to 76.6 in School 5. The prevalence of stunting was 2.5% in School 1 compared to 55.6% in School 5 at first observation. In schools 4 and 5, there are few observations at grades 7 and 9, which is likely related to few students continuing in education beyond grade 6 from lower socioeconomic backgrounds. There was a high level of clustering in the data, whereby 55.6% of variance in IQ was explained by between school differences, 27% was due to between individual differences, and 17.4% of variance was explained by differences between measurement occasion within individuals.

**Table 1:** Descriptive statistics.

		School 1	School 2	School 3	School 4	School 5
Total participants	N	3758	10,197	1979	2972	3818
Sex						
Male	N (%)	1965 (52.3)	6146 (60.3)	1135 (57.3)	1695 (57)	2129 (55.8)
Female	N (%)	1793 (47.7)	4051 (39.7)	844 (42.7)	1277 (43)	1689 (44.2)
IQ						
Value	Mean (SD)	107.2 (10.4)	100.1 (10.1)	85.4 (11.1)	81.5 (10.8)	76.6 (10.5)
Number of observations	Mean (SD)	3.2 (1.5)	3.0 (1.5)	1.8 (1.0)	2.1 (1.1)	2.2(1.2)
Age range of observations	Mean (SD)	6.6 (3.1)	6.1 (3.0)	2.7 (2.3)	3.1 (2.1)	3.9 (2.7)
Birth year	Mean (SD)	1978 (7)	1978 (7)	1972 (4)	1982 (5)	1980 (6)
	Range	1962–1992	1961–1993	1961–1981	1969–1993	1964–1992
Observations by school year						
Grade 1	N (%)	2552 (21.1)	5598 (18.1)	970 (27.0)	1771 (28.7)	2720 (32.9)
Grade 2	N (%)	2521 (20.9)	5964 (19.3)	755 (21.1)	1528 (24.8)	1965 (23.8)
Grade 4	N (%)	2460 (20.4)	5910 (19.1)	763 (21.3)	1448 (23.5)	1685 (20.4)
Grade 6	N (%)	2332 (19.3)	5748 (18.6)	648 (18.1)	1422 (23.1)	1235 (14.9)
Grade 7	N (%)	2146 (17.8)	6401 (20.7)	397 (11.1)	0	617 (7.5)
Grade 9	N (%)	63 (0.5)	1269 (4.1)	54 (1.5)	0	44 (0.5)
Observations by OLMAT level						
Elementary I Level Form J	N (%)	2552 (21.1)	5598 (18.1)	970 (27.0)	1771 (28.7)	2720 (32.9)
Elementary I Level Form K	N (%)	2521 (20.9)	5964 (19.3)	755 (21.1)	1528 (24.8)	1965 (23.8)
Elementary II Level Form K	N (%)	2460 (20.4)	5910 (19.1)	763 (21.3)	1448 (23.5)	1685 (20.4)
Intermediate Level Form J	N (%)	4478 (37.1)	12,149 (39.3)	1045 (29.1)	1422 (23.1)	1852 (22.4)
Advanced Level Form J	N (%)	63 (0.5)	1269 (4.1)	54 (1.5)	0	44 (0.5)
Height cm	Mean (SD)	140.0 (1.5)	138.8 (1.5)	130.0 (1.4)	126.7 (1.3)	126.4 (1.3)
Height Z-score	Mean (SD)	−0.2 (1.0)	−0.5 (1.0)	−1.4 (1.0)	−1.7 (0.9)	−2.1 (0.9)
Stunting prevalence (at first observation)	N (%)	93 (2.5)	590 (5.8)	587 (29.7)	1165 (39.2)	2122 (55.6)

**Fig. 1:**
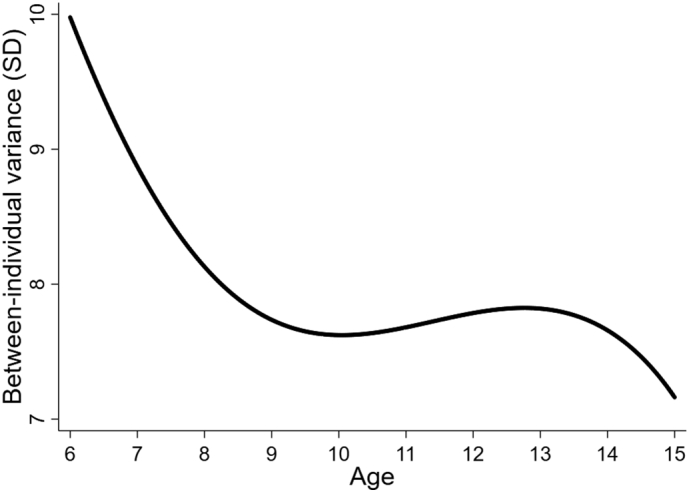
Between-individual variance^1^. ^1^ This curve is a function of the level 2 random effects (i.e. the differences between students).

Estimates of the multilevel model are presented in Table 2. Overall model fit was determined to be good according to diagnostic plots and investigation of the level 1 random effects (i.e., residuals). Level 1 variance in all schools was lower than 40, which equates to a standard deviation of approximately 6.3 IQ points (i.e., sqrt (40) = 6.3). Level 1 variance did however tend to increase across the schools, thereby demonstrating greater within-person variability in lower (compared to higher) SES schools. The constant, age, and age^2^ terms together produced a sample-average trajectory in which IQ declined from approximately 103 at age 6 years to 90 at age 12 years (data not shown). These three terms had random effects at level 2 & 3, thereby allowing each individual and school to have their own trajectory. Fig. 1 illustrates how the standard deviation of the individual trajectories was approximately 10 IQ points at age 6 years decreasing to 7 at age 15 years, that is, the differences in IQ between all participants at all schools decreased as they got older.

**Table 2:** Estimates from the multilevel model applied to 60,986 IQ observations in 22,724 children.

		Estimate	95%CI		P
Fixed Part	Constant	89.9	80.2	99.5	<0.001
	Age	−0.8	−1.0	−0.6	<0.001
	Age^2^	0.4	0.3	0.5	<0.001
	Birth year	0.14	0.12	0.16	<0.001
	Age x Birth year	−0.014	−0.020	−0.010	<0.001
	Height Z-score if <0	1.4	0.7	2.1	<0.001
	Height Z-score if ≥0	0.3	0.001	0.5	0.049
	Sex				
	Male (referent)	–	–	–	
	Female	−1.6	−1.9	−1.4	<0.001
Random Part	Variance				
Level 3: School	Constant	120.2	−28.9	269.4	
	Age	0.06	−0.02	0.1	
	Age^2^	0.01	−0.004	0.03	
	Height Z-score if <0	0.6	−0.2	1.4	
Random Part	Variance				
Level 2: Individual	Constant	60.0	57.2	60.7	
	Age	0.5	0.4	0.6	
	Age^2^	0.07	0.06	0.08	
	Covariance				
	Constant, Age	0.8	0.6	1.0	
	Constant, Age^2^	−0.09	−0.2	0.02	
	Age, Age^2^	−0.2	−0.2	−0.1	
Random Part	Variance				
Level 1: Visit	School 1	26.1	25.1	27.0	
	School 2	24.1	23.5	24.7	
	School 3	30.9	28.7	33.2	
	School 4	39.9	37.8	42.0	
	School 5	38.0	36.3	39.7

**Fig. 2:**
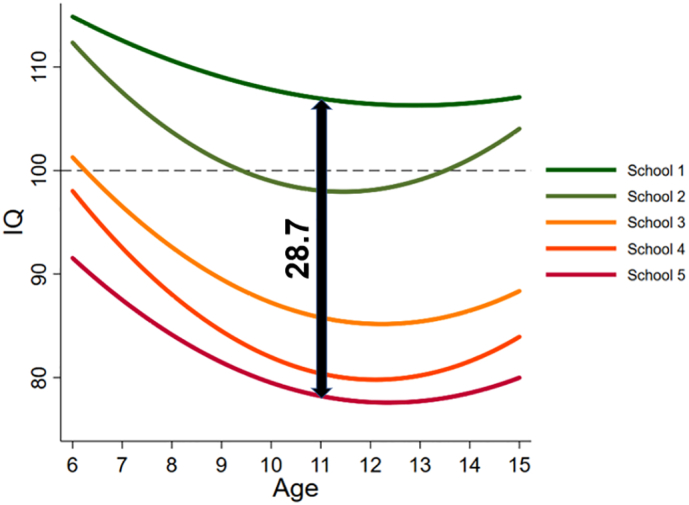
IQ trajectories across age according to school.

Fig. 2 shows that the IQ trajectories for each school were clearly ranked, with lower socioeconomic status schools having lower trajectories across the entire age range. At age 11 years, for example, the trajectory for School 1 was estimated to be 28.7 IQ points higher than the trajectory for School 5, although the estimate was imprecise with the 95% CI overlapping with zero (*p* > .05), Table 2).

**Fig. 3:**
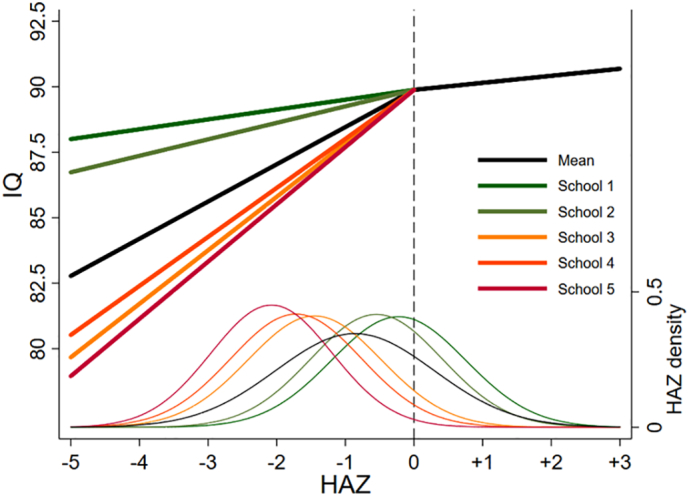
Relationships of height *Z*-scores with IQ according to school.

In the full sample, a one-unit increase in HAZ was associated with a 1.42 (0.72, 2.11) unit higher IQ if HAZ was <0 but only a 0.3 (0.001, 0.5) unit higher IQ if HAZ was ≥0. As shown in Fig. 3, the first HAZ term had a stronger relationship with IQ in the lower SES schools (3–5) than in the higher SES schools (1–2) although, again, the 95% CI overlaps with zero. The reality of UVG database is that an investigation of school effects for the second height term is not feasible, due to the low number of individuals in these groups with HAZ > 0, as the HAZ density curves show (Fig. 3).

**Fig. 4:**
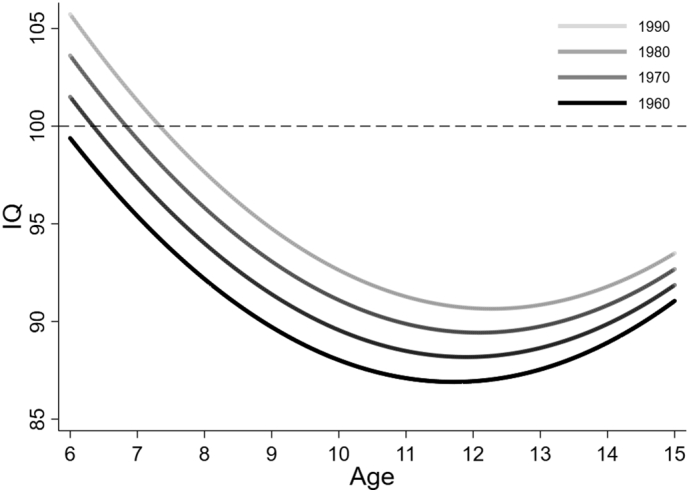
Predicted IQ trajectories across age according to birth year.

With each birth year increase, IQ at age 11 years increased by 0.14 (95% CI 0.12, 0.16) units, although this effect size attenuated slightly across adolescence due to the negative age X birth year term. Fig. 4 illustrates this Flynn effect by showing predicted IQ trajectories for birth years – 1960, 1970, 1980, and 1990. During model development, we tested interactions between the HAZ terms and birth year but found no evidence that the height inequality in IQ had changed over time. During model development, we also tested by allowing birth year have a random effect at level 3 but found no evidence that the Flynn effect differed across schools. This also means that the inequality between schools has remained consistent over time.

## 4 Discussion

This study documents difference in childhood-to-adolescence IQ trajectories in Guatemala City related to school (a proxy for community-level conditions) and HAZ (a proxy for individual-level conditions), the latter particularly among shorter than average students and in lower SES schools. We also observed the Flynn effect (approximately 1.4 points per decade at age 11) but did not find any evidence that the documented differences between schools had changed over time.

The IQ trajectory in the present study had a U-shape and highlighted a ‘dip’ in IQ at around age 11. A very similar pattern has been previously reported for a large cohort of UK girls by UK Twins Early Development Study (TEDS), including variation in the shape of the trajectory by SES (high SES individuals showed a shallower slope in the IQ by age curve) (von Stumm & Plomin, 2015). The UK boys investigated by the same study showed an inverse pattern, where IQ increased around age 11 before a decline in puberty. We found no evidence of a sex difference in IQ by age trajectories in the Guatemalan data. The decreasing IQ from childhood to adolescence could reflect changes in brain development, however this remains speculative as we do not have data to investigate this.

Between-individual variance in IQ scores was highest at younger ages (6–7 years). The reason for this is unclear but could relate to the more variable attention span of younger children, students becoming more alike after attending the same school for several years, or perhaps reflect a more fluctuating motivation to take the test at young ages. Within-individual variance in IQ scores was greatest in the lower SES schools (that also had lowest average IQ). Lower fluid intelligence is known to correlate with higher intraindividual variability in repeated cognitive tasks (Galeano Weber, Dirk, & Schmiedek, 2018). The reason for these findings could be that the motivation and preparedness of individuals to take part in tests was higher and more consistent in the higher SES schools, where students are likely to have been more used to standardised testing. Alternatively, as fewer students in the lower SES schools continue in education beyond grade 6, they may exhibit less of the stabilization in IQ which is often assumed to take place post-puberty (Tucker-Drob & Briley, 2014). This may be because those lower SES students who do continue their schooling are selected for school performance and cognitive ability and may experience the well-known positive feedback effect between more years of education and higher IQ (Sauce & Matzel, 2018). A complimentary explanation may be the ‘multiplier effect’ proposed by Dickens and Flynn (2001), whereby those with a greater IQ tend to seek stimulating environments that further increase IQ, such as further schooling.

The magnitude of the SES inequality in the present study (measured by school) was nearly two IQ standard deviations (28.7 IQ points) between schools 1 and 5. In the UK, SES related inequality in IQ, albeit not proxied by school, is only 6 IQ points at 2 years of age, and 15–17 points at age 16 (von Stumm & Plomin, 2015). One reason for this difference may be that children and their parents in the UK benefit from access to both universal health care and higher quality state-sponsored education where school meals are available to disadvantaged students. In Guatemala, overall economic and health disparities are extremely high and there is relatively little investment by the state into basic health services (PAHO, 2016). State investment to education is only 2.8% of GDP, which is the lowest in all of Latin America (OECD, 2015; UNDP, 2013). Thus, the students attending the public, lower SES schools in the present study are likely to be disadvantaged in terms of both quality of education they receive, as well as overall resources available to them (e.g. nutrition, health care). In the present study the IQ trajectories of each school were relatively similar in shape, and the differences between schools remained relatively stable across ages, which suggests that attending a specific school was not associated with increased or decreased IQ difference. This is in contrast with work from Germany that found a larger positive effect of schooling on IQ in ‘academic’ track compared to vocational track secondary schools (Becker et al., 2012). Germany, like the UK, provides a relatively high standard of living to all citizens and SES differences in economic and health status are small compared to Guatemala. The German findings may reflect school selection based on IQ of the students, while the Guatemalan findings may reflect IQ (and HAZ) impairment due to poverty and poor-quality schools for the poor. Therefore, with more equal conditions and opportunities for the Guatemalan students of the lower SES we might see a substantial decrease in the difference in measured IQ between the schools.

A meta-analysis of studies from LMICs found a positive, linear association between HAZ at under age 2 years, and IQ measured at ages 5–11 (Sudfeld et al., 2015). The present study found a strong positive association between HAZ at ages 6–15, and IQ if HAZ was lower than 0, and no strong association if HAZ was greater than 0. The only previous work illustrating a similar association comes from the UK's National Child Development Study (1958 birth cohort) (McManus & Mascie-Taylor, 1983). In the 1958 cohort, the association between general cognitive ability (‘g’) and HAZ was described as quadratic with the slope clearly reduced at HAZ > 0. This implies that in impoverished environments there may be a common mechanism that can result in both linear growth faltering (short stature for age) and impaired cognitive development. This conclusion is supported by the moderating role of school on the IQ-HAZ association, as we found that the association at HAZ < 0 was stronger in the lower SES schools, (although the estimates were imprecise with the 95% CI overlapping with zero). The effect of school/SES is hard to separate from the effect of height, as the lower SES schools contain shorter individuals, but overall it seems that in poorer environments HAZ and IQ are more strongly associated than in more advantaged settings. A similar finding has been reported for child reading skill in India, whereby the height–achievement slope for participants in the India Human Development Survey, a representative sample of 40,000 households, was found to be more than twice as steep than for US children (Spears, 2012). The findings for Guatemala and India may indicate that individuals of short height in higher SES groups may be ‘just short’, or alternatively, better buffered against the broader negative correlates of shorter height. Alternatively, as the range of HAZ < 0 for Schools 1 and 2 is more restricted than for Schools 3–5, the association of IQ and HAZ in the higher SES schools may be attenuated.

The average IQ of the UVG Study sample increased over time, at age 11 average IQ was 4.2 points higher in 1990 compared to 1960. The per decade increase was 1.4 points, less than the average of 2.3 points reported by a recent meta-analysis of the Flynn effect for all US and UK samples measured between 1951 and 2010 (Trahan et al., 2014). The effect is less well documented for LMICs but evidence from South Africa suggests gains of 2.5 points per decade for ‘White’ individuals born between 1890 and 1985, while Brazil has seen an increase of 2.3 points per decade in 11-year olds from Belo Horizonte (Colom et al., 2007; Te Nijenhuis, Murphy, & van Eeden, 2011). The cause for the Flynn effect is thought to be multifactorial, and related to increased standard of living, more and better education, and improvements in the nutritional and disease environment (Rindermann, Becker, & Coyle, 2017; Williams, 2014). There were major social, political and economic upheavals in Guatemala over the course of the UVG Study, including the 40-year civil war which ended in 1996 (Tomuschat et al., 1999). Despite the war, the country did experience general economic growth between 1960 and 1985 (Trading Economics, 2018). These wider environmental factors may have influenced the secular IQ increase of urban Guatemalan children. The internal conflict may have introduced a negative trend in in both HAZ and IQ, while the economic growth may have caused a trend toward higher HAZ and IQ. These conflicting factors might help explain why the size of Flynn effect is approximately 1 IQ point lower in Guatemala than elsewhere. The Flynn effect was larger at younger ages, as highlighted by the negative age and birth year interaction term. This finding is support by evidence that the heritability of IQ is lowest at younger ages (Tucker-Drob & Briley, 2014), that is, the environment may have a stronger influence on IQ at younger ages. The present study did not find evidence for change in inequality in IQ over time in Guatemala, which means that the Flynn effect was the same regardless of school attended, or individual height. This is in contrast with the finding that in some populations, including the US and Spain, the effect has been accompanied by decreasing variability in IQ scores due to improved performance of particularly the lowest end of the IQ distribution, thus decreasing the overall IQ inequality (Pietschnig & Voracek, 2015).

Through analysis of a large longitudinal dataset, collected over 40 years, this study employed a multilevel modelling framework to simultaneously account for variance at within-individual, between-individual, and group level. The sample is representative of a broad socioeconomic status range and includes individuals of both Maya and Ladino ethnicity. Our findings should not be wrongly used to reinforce negative stereotypes about the Maya of Guatemala being short and of low cognitive ability, which are commonly expressed by popular media and some researchers both in Guatemala and abroad. Instead, our findings should be used to demonstrate how decades of social, economic, and political inequality and oppression impact on the low SES population of Maya and Ladinos in Guatemala.

A limitation of this study is the possible influence of the repeated IQ testing in the same sample. There is a large body of literature on retest effects, which highlights that cognitive test performance is increased when the same test is taken twice, and sometimes even when using different instruments (Scharfen, Peters, & Holling, 2018). We believe this limitation has relatively little impact on our findings, as the typical testing interval in the UVG Study was two years, which is considered to be long enough to result in negligible retest effect (Scharfen et al., 2018). Further, the tests increased in difficulty each testing event. Another limitation of our study is that there is no psychometric information about the quality of the OLMAT in the context of Guatemala, and the IQ scores were obtained using US reference norms. If there are consistent differences in the development of IQ at different ages between the countries, then this could impact the interpretation of our findings. The performance of children attending School 5, particularly in the first test, may have been influenced by the tests being conducted in the school language Spanish, not in their home language Kaqchikel Maya. But, as the test taken in the first year did not require reading skill, the impact of this on test performance should be minimal. An additional limitation is that due to the study design, we do not know if the differences reported are due to the education being received or are driven by other characteristics of the children that attend each school.

## 5 Conclusion

Shorter children from disadvantaged schools in Guatemala City suffer the most from inequality in IQ, possibly reflecting the damaging effects of poor early life environments both for growth in stature and cognitive development.
